# A Metagenomic and in Silico Functional Prediction of Gut Microbiota Profiles May Concur in Discovering New Cystic Fibrosis Patient-Targeted Probiotics

**DOI:** 10.3390/nu9121342

**Published:** 2017-12-09

**Authors:** Pamela Vernocchi, Federica Del Chierico, Andrea Quagliariello, Danilo Ercolini, Vincenzina Lucidi, Lorenza Putignani

**Affiliations:** 1 Unit of Human Microbiome, Bambino Gesù Children’s Hospital, IRCCS, Viale San Paolo 15, 00146 Rome, Italy; pamela.vernocchi@opbg.net (P.V.); federica.delchierico@opbg.net (F.D.C.); andrea.quagliariello@opbg.net (A.Q.); 2 Department of Agricultural Sciences, Division of Microbiology, University of Naples Federico II, Via Università 100, Portici, 80055 Napoli, Italy; danilo.ercolini@unina.it; 3 Cystic Fibrosis Unit, Bambino Gesù Children’s Hospital, IRCCS, Piazza Sant’ Onofrio 4, Rome 00165, Italy; vincenzina.lucidi@opbg.net; 4 Unit of Parasitology Bambino Gesù Children’s Hospital, IRCCS, Piazza Sant’ Onofrio 4, Rome 00165, Italy

**Keywords:** cystic fibrosis, gut microbiota profiling, KEGG prediction-tailored probiotic design

## Abstract

Cystic fibrosis (CF) is a life-limiting hereditary disorder that results in aberrant mucosa in the lungs and digestive tract, chronic respiratory infections, chronic inflammation, and the need for repeated antibiotic treatments. Probiotics have been demonstrated to improve the quality of life of CF patients. We investigated the distribution of gut microbiota (GM) bacteria to identify new potential probiotics for CF patients on the basis of GM patterns. Fecal samples of 28 CF patients and 31 healthy controls (HC) were collected and analyzed by 16S rRNA-based pyrosequencing analysis of GM, to produce CF-HC paired maps of the distribution of operational taxonomic units (OTUs), and by Phylogenetic Investigation of Communities by Reconstruction of Unobserved States (PICRUSt) for Kyoto Encyclopedia of Genes and Genomes (KEGG) biomarker prediction. The maps were scanned to highlight the distribution of bacteria commonly claimed as probiotics, such as bifidobacteria and lactobacilli, and of butyrate-producing colon bacteria, such as *Eubacterium* spp. and *Faecalibacterium prausnitzii.* The analyses highlighted 24 OTUs eligible as putative probiotics. Eleven and nine species were prevalently associated with the GM of CF and HC subjects, respectively. Their KEGG prediction provided differential CF and HC pathways, indeed associated with health-promoting biochemical activities in the latter case. GM profiling and KEGG biomarkers concurred in the evaluation of nine bacterial species as novel putative probiotics that could be investigated for the nutritional management of CF patients.

## 1. Introduction

Cystic fibrosis (CF) is an autosomal recessive condition occurring among people with European origins, which is caused by mutations in the cystic fibrosis transmembrane conductance regulator (CFTR) gene. The CFTR mutation leads to the failure or the absence of functional CFTR proteins at the apical membrane of epithelial cells in several body systems [1]. The CFTR protein, in addition to functioning as a chloride channel, can also affect bicarbonate transport. Protein mutations cause the formation of viscous and dehydrated mucus followed by the establishment of aberrant mucosa in the lungs and digestive tract. This condition increases the risk of recurrent and chronic pulmonary infection and inflammation, pancreatic insufficiency (PI), CF-related liver disease, and diabetes [2].

The recurrent destructive airway infections, determined by the progressive inflammatory lung diseases, represent the principal cause of mortality, morbidity, and altered quality of life in CF patients, resulting in respiratory failure in 90% of patients with CF [3]. 

To reduce pulmonary exacerbation, patients are subjected to an antibiotic therapy which leads to the modification of the gut microbiota (GM) [4]. The CFTR mutations also lead to the alteration of intestinal permeability, determining an impaired composition and function of the intestinal barrier. The production of immune mediators is altered alongside with mucosal inflammation, triggering an increase in the concentrations of fecal calprotectin and rectal nitric oxide [5]. The mutations can also affect the body’s endocrine, neural, and immune systems [6]. This clinical status also leads to a compromised nutritional status associated with the severity of CF disease, which unfortunately affects the quality of life and life expectancy [7]. 

The maintenance of an optimal nutritional status may ameliorate the quality of life of CF patients, especially during rehabilitation programs and therapies targeting the respiratory infections [8]. GM modulation induced by nutritional intervention may have implications in the management of CF-related malnutrition and comorbidities, since diet is perhaps the most modifiable factor that shapes microbiota profiles [9]. 

The diet-driven functional evolution of the GM has been thoroughly discussed in mammalian species, starting at neonatal age [10,11]. The maintenance of microbiota eubiosis seems to contribute to the prevention and clarification of complex disease phenotypes [12]. In particular, the administration of probiotics also contributes to GM eubiosis maintenance and restoration in CF patients [13].

Probiotics are defined as “live microorganisms that, when administered in adequate amounts, confer a health benefit on the host” [14]. They colonize the intestine and affect either microbiota composition or function, acting on the host epithelial and immunological responses [15], reducing intestinal inflammation, and hence improving the intestinal functions at clinical and biochemical levels [16,17,18] even when they were altered by an antibiotic therapy [17]. Probiotics have been used with positive outcomes in childhood gastroenteritis, atopic diseases, and *Helicobacter pylori* infection [19]. Specifically, the administration of *Lactobacillus* GG can decrease the incidence of exacerbations and reduce the intestinal inflammation in CF patients, as reported by Bruzzese [20]. Potential mechanisms of action for probiotics in CF include their influence on gut motility and intestinal barrier function and the inhibition of pathogenic bacteria colonization [2]. 

There is evidence that probiotic administration in these patients reduces pulmonary exacerbation rate and hospital admission [21]. Clinical trials on probiotic administration in CF patients are on the rise [17,22], but there is no evidence of an optimal patient-tailored probiotics regimen to be administered for this chronic disease. 

The aim of this study was to evaluate the distribution of *Bifidobacterium* spp., *Lactobacillus* spp., *Eubacterium* spp., and *Faecalibacterium prausntizii* in the GM of CF and healthy subjects. We focused on species commonly claimed as targets for the design of novel probiotics [23]. According to a targeted metagenomics analysis and functional prediction of related Kyoto Encyclopedia of Genes and Genomes (KEGG) pathways, we propose potential probiotics species for CF management.

## 2. Material and Methods

### 2.1. Patients

This study was conducted on 28 consecutive CF patients aged 1 to 6 years (average age 3.5 years, SD ± 1.69; 11 males and 17 females), recruited at the Cystic Fibrosis Unit of the Bambino Gesù Children’s Hospital (OPBG, Rome, Italy) over one year (2012). The diagnosis of CF was made on the basis of the results of a pathological sweat test (chloride > 60 mmol/L, reference value), as described by Gibson and Cooke [24], or by the presence of two CF-causing mutations in the CFTR gene [25].

The study protocol was approved by the OPBG Ethics Research Committee (protocol No. 534/RA), and was conducted in accordance with the Declaration of Helsinki (as revised in Seoul, Korea, October 2008). A signed informed consent was obtained from the parents of the enrolled subjects. The patients were age-matched with 31 healthy controls (HC) screened by means of a survey of the OPBG Human Microbiome Unit on pediatric gut microbiota programming.

Inclusion criteria for HC were: absence of any inflammatory, infectious, and chronic diseases at the time of the microbiota analysis and no antibiotic and pre-probiotic intake in the previous two months.

For CF patients, the inclusion criteria consisted of being recruited under clinical stability (i.e., absence of infectious exacerbation of pulmonary symptoms) and no pre-probiotic intake in the previous two weeks.

### 2.2. Anamnestic and Laboratory Features

Age, gender, and body mass index (BMI) (for patients over 2 years of age) or Z-score (Weight/Length (W/L) of patients under 2 years of age) were collected for both CF patients and HC, whereas sweat chloride test values, pancreatic status (PS), and antibiotic data for chronic regimen were collected only for CF patients (Table 1). 

### 2.3. DNA Extraction and Next Generation Sequencing (NGS) Analysis

Fecal samples (59) were collected from each subject during clinical examination and stored until metagenomics analysis. The genomic DNA was isolated using the QIAamp DNA Stool Mini Kit (Qiagen, Hilden, Germany). The V1–V3 region (520 bp) of the 16S ribosomal RNA locus was amplified for pyrosequencing analysis using a 454-Junior Genome Sequencer (Roche 454 Life Sciences, Branford, CT, USA) according to Del Chierico et al. [26]. The nucleotide barcodes, added in forward primers, were composed of 8 unique nucleotides (Roche 454 Life Sciences). The polymerase chain reactions were performed using Hi-Fi Polymerase Chain Reaction (PCR) Taq polymerase (FastStart™ High Fidelity PCR System, dNTPack, Roche Diagnostics, Mannheim, Germany), guaranteeing high specificity, sensitivity, and accuracy during PCR amplification. 

### 2.4. Statistical Analysis 

Reads were analyzed with Qiime 1.8 (Quantitative Insights Into Microbial Ecology, http://qiime.org/1.4.0/) using the default pipeline [27]. After demultiplexing, reads with an average quality score lower than 25, shorter than 300 bp, and with an ambiguous base calling were excluded from the analysis to guarantee a higher level of accuracy in terms of detection of the operational taxonomic units (OTUs). Sequences that passed the quality filter were denoised [28], and singletons were excluded. The denoised sequences *were chimera-checked by identify_chimeric_seqs.py*. 

To characterize the taxonomic structure of the samples, the sequences were organized into OTUs by clustering at a threshold of 97% pairwise identity and by classifying the representative sequences using the Greengenes 13_8 database [29]. The representative sequences were submitted to PyNAST for sequence alignment [30] and to UCLUST for sequence clustering [31]. 

The OTU Kruskal–Wallis tests were performed by QIIME software (http://qiime.org/1.4.0/) using “group_significance.py” script [32]. The Kruskal-Wallis test was performed on OTU distribution with False Discovery Rate (FDR) correction (*p*-value ≤ 0.1). To gain more insight into the metagenomics-based function of the microbiome of the CF patients and HC, the Phylogenetic Investigation of Communities by Reconstruction of Unobserved States (PICRUSt) v1.1.0 tool was used [33], and the resulting function prediction was analyzed using the HUMAnN2 v0.99 program to get KEGG pathways (http://huttenhower.sph.harvard.edu/humann2) [34]. To find possible OTUs and KEGG biomarkers associated with CF and HC, a linear discriminant effect size (LEfSe) analysis was performed [35] with the α value of the statistical test equal to 0.05 and the logarithmic Linear Discriminant Analysis (LDA) score threshold equal to 2.0.

## 3. Results 

### 3.1. Putative Probiotic Distribution in the GM Profiles

By targeted metagenomics, a total of 316,000 reads was obtained with an average of 5356 reads/sample and an average length of 487 bp. Genus-level comparisons were performed on 24 OTUs belonging to *Bifidobacterium* spp., *Lactobacillus* spp., *Eubacterium* spp., and *F. prausnitzii* chosen from a total dataset of 165 OTUs, considering their putative probiotic role.

**Figure 1:**
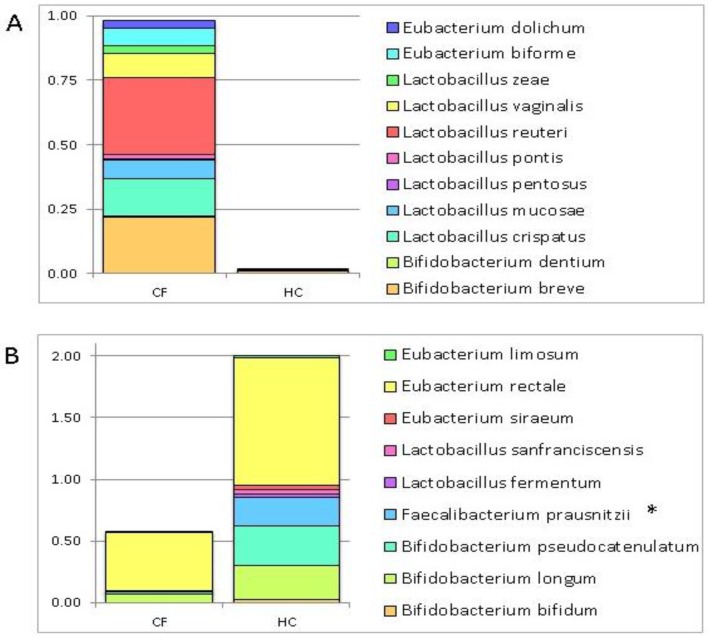

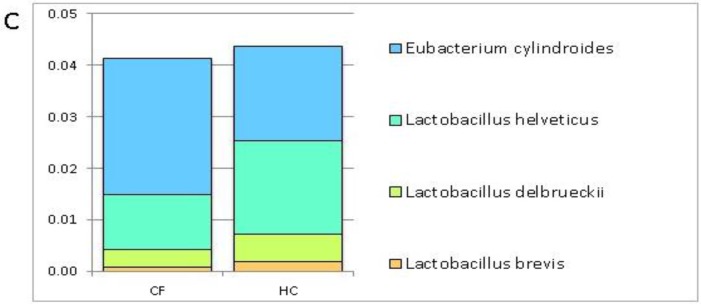
Histograms of the relative abundance of 24 selected operational taxonomic units (OTUs) in the gut microbiota (GM) patterns of cystic fibrosis (CF) patients and healthy controls (HC). These OTUs were chosen for their putative probiotic role. The histograms show the relative abundance of the searched putative probiotic bacteria scanned through the GM patterns of the CF patients and HC. (**Panel A**): 9 OTUs prevalently distributed in the GM profile of the CF subjects (relative abundance > 0.001); (**Panel B**): 11 OTUs prevalently distributed in the GM profile of the HC (relative abundance > 0.02). *Fecalibacterium prausnitzii* shows a statistically significant value False Discovery Rate (FDR) adjusted *p* value ≤ 0.1); (**Panel C**): 4 OTUs comparably distributed in the GM profiles of the CF patients and HC.

The profiling of targeted metagenomic sequencing pointed out a distribution of 11 bacterial species prevalently associated with the GM of the CF patients (Figure 1, Panel A), and 9 species prevalently associated with the GM of the HC (Figure 1, Panel B) (Table 2). The Kruskal–Wallis test identified a statistically significant difference for *F. prausnitzii* distribution between CF patients and HC, highlighting a higher relative abundance in HC.

On the contrary, the remaining 4 OTUs, namely, *Lactobacillus brevis*, *L*. *delbrueckii*, *L*. *helveticus,* and *Eubacterium cylindroides* were comparably distributed in the GM profiles of the CF patients and HC (Figure 1, Panel C). 

### 3.2. Metabolic Pathways of Probiotics

To better define the metabolic role of the detected putative probiotic species, a supervised comparison of CF patients’ and HC’s KEGGs was inferred by LEfSe on the 24 OTU matrix.

**Figure 2:**
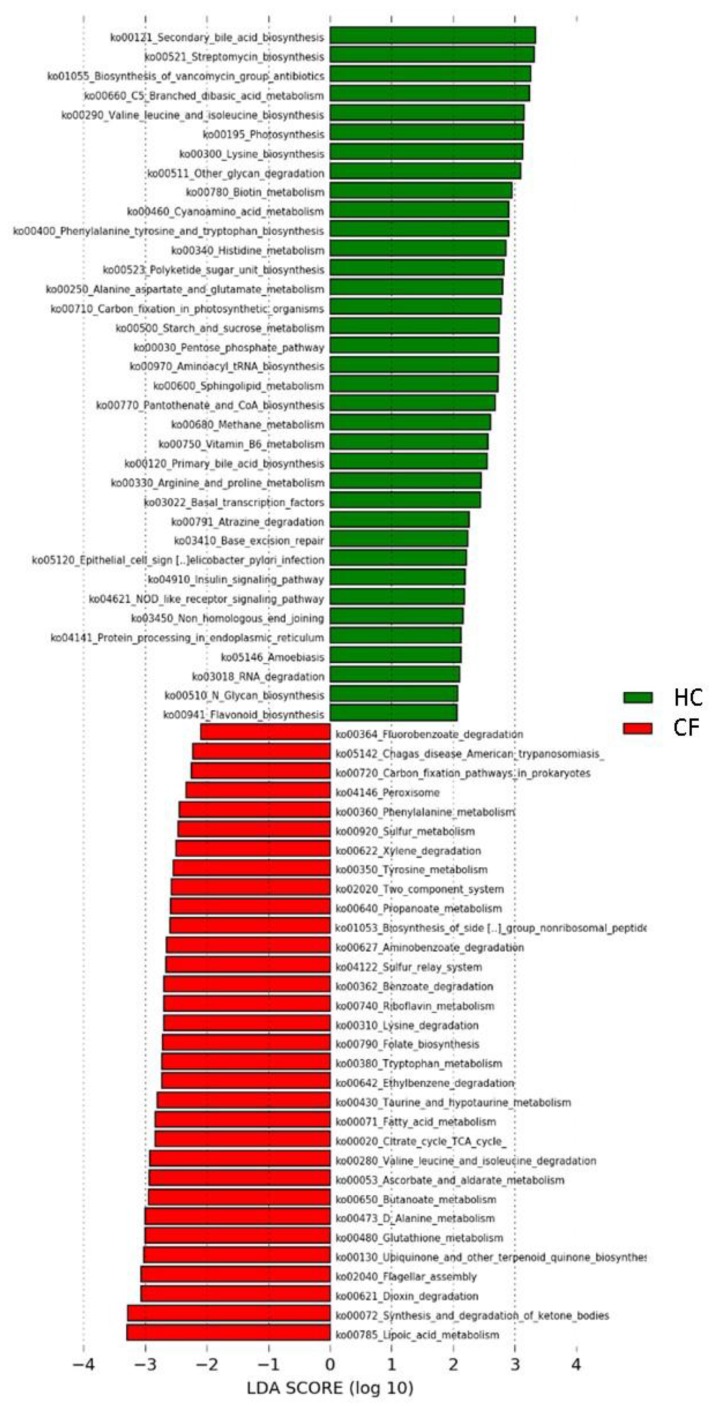
Kyoto Encyclopedia of Genes and Genomes (KEGG) biomarkers inferred from the whole set of 24 OTUs of putative probiotic bacteria scanned through the GM patterns of CF patients and HC subjects. A linear discriminant effect size (LeFse) analysis was performed (α = 0.05, logarithmic Linear Discriminant Analysis (LDA) score threshold = 2.0).

The predicted microbial function highlighted differences in metabolic pathways associated with the 24 selected OTUs (Figure 2). In particular, 24 pathways resulted associated with CF and 39 with HC (Table 3).

## 4. Discussion

### 4.1. Putative Probiotic Distribution in the GM Profiles

Bifidobacteria and lactobacilli are recognized as beneficial bacteria for their intrinsic probiotic features [23]. *Eubacterium* spp. may aid in the digestion, the absorption, or both of food ingredients and minerals, especially under malnutrition conditions usually occurring in CF because of nutrient absorption defects [36]. More generally, *F. prausntizii* has been recently proposed to provide high butyrate production in the gut [37].

Among the bacteria associated with the CF gut profiles (Table 2), some are actually linked to different pathologic conditions. Indeed, *Bifidobacterium dentium* was detected in the oral cavity in association with dental caries [38], while *Eubacterium dolichum* was associated with frailty in the elderly, a condition that represents the biggest problem associated with population aging [39]. *Lactobacillus mucosae* was detected in the microbiota of short bowel syndrome patients [40]. Other bacterial strains identified for CF patients were associated with metabolic disorders, such as high total cholesterol and low-density lipoprotein levels (*Eubacterium biforme*) [41], obesity (*Lactobacillus reuteri*) [42], and nonalcoholic fatty liver disease (NAFLD) (*L. zeae* and *L. vaginalis*) [24].

Unlike the aforementioned negative roles of the previous reported bacteria in human health, *B. breve* is considered a commensal or even a health-promoting microorganism [43] because it improves symptoms in necrotizing enterocolitis [44] and atopic dermatitis [45], as well as those associated with HIV-induced damages [46]. Moreover, *B. breve* shows antimicrobial activity [47], induces innate immune responses, and has anti-inflammatory effects [48]. Also, *L. pentosus* was reported to ameliorate colitis in the aged rodent by inhibiting the activation of nuclear factor (NF)-κB, activator protein 1 (AP1), and mitogen-activated protein kinases (MAPKs) [49]. 

No role in the human GM or putative effects as probiotics have been reported for *L. crispatus* and *L. pontis*. 

The GM in the HC group seemed to be enriched in species involved in gut integrity and mobility, digestion of specific dietary compounds, and immune system modulation

Indeed, in the research of Kanauchi and co-workers [50], *E. limosum* was presented as an important probiotic candidate for its short-chain fatty acid (SCFA) production, role in maintaining and enhancing mucosal integrity, and anti-inflammatory properties in the intestinal mucosa [50]. Moreover, Bruzzese et al. found that both *E. rectale* and *F. prausnitzii* were reduced in the GM of CF children compared to HC, confirming our results [51].

*Eubacterium siraeum* is able to degrade wheat bran, contributing to the beneficial effects of cereal fiber in human health through their impact on the GM [52].

*L. sanfranciscensis* is generally considered the most important lactic acid bacterium in the fermentation of rye and wheat sourdoughs [53]. The strain *L. sanfranciscensis* LBH1068, tested in an induced chronic colitis mouse model, improved mouse health by reducing weight loss, decreasing gut permeability, and modulating cytokine production [54].

In addition, *L. fermentum* demonstrated intestinal anti-inflammatory effects in the model of sodium dextran sulfate-induced colitis in mice. Among the mechanisms proposed, *L. fermentum* restored GM composition and modulated the altered immune response by preserving intestinal barrier integrity, decreasing pro-inflammatory cytokine production, and modulating the expression of Th1-, Th17- and T_reg_-related cytokines [55]. 

The study of Moya-Pérez and colleagues demonstrated that *B. pseudocatenulatum* modulated immune cell infiltration and inflammation in the gut in obesity [56].

A reduction in bifidobacteria in CF, especially *B. longum*, was already reported by Duytschaever et al. [57]. High richness of bifidobacteria species was positively correlated with the maturation of the mucosal immune system [58]. *B. longum* was found to be an inhibitor of rotavirus, the predominant cause of sporadic diarrhea in infants [59]. A recent study has demonstrated that acetate produced by *B. longum* acts as an essential cosubstrate for butyrate production and for *E. rectale* growth [60].

*B. bifidum* and *B. longum* possess numerous pathways involved in the catabolism of human milk oligosaccharides (HMO) and may also consume carbohydrates released by other bacteria [61]. *B. bifidum* and *B. longum* were described as being more abundant in healthy subjects compared to NAFLD patients, suggesting a protective and beneficial role also in obesity and NAFLD [62]. 

Finally, each of these microbial species, especially those lacking in patients’ GM (Table 2, HC-related species), could be considered suitable for the design of CF patient-tailored probiotics.

### 4.2. Metabolic Pathways of Probiotics

To evaluate the microbial metabolic and functional KEGG pathways of the chosen putative probiotic species, a supervised comparison of CF patients’ and HC’s KEGGs was performed by LEfSe (Table 3). Pathways associated with fatty acid biosynthesis, metabolism, and synthesis and degradation of ketone bodies were significantly associated with CF patients, as already described by Fouhy et al. [63]. This increase in fat metabolism probably occurs as a result of a combination of factors, including a reduced intestinal absorption and an altered GM in CF patients. Reduced fat absorption is one reason why most CF patients are traditionally prescribed a high-fat diet to ensure adequate weight maintenance. Moreover, it is possible that the altered GM might contribute to the increase in fat metabolism [63].

On the contrary, primary and secondary bile acid biosynthesis pathways were associated with the GM of HC. It is known that CF patients have a variety of intestinal abnormalities in bile acid metabolism at the intestinal level, including increased fecal bile acid losses, reduced bile acid pool size, and duodenal bile acid concentration [64]. These abnormalities appear to be associated with exocrine pancreatic insufficiency and steatorrhoea. Indeed, improvement of bile abnormalities with amelioration in fat malabsorption was reported after pancreatic enzyme therapy [64].

Pathways involved in xenobiotic metabolism have been significantly observed in the GM pattern of CF patients, including benzoate, fluorobenzoate, dioxin, xylene, aminobenzoate, and ethylbenzene degradation pathways. Bacterial pathways involved in xenobiotic metabolism were also observed by Fouhy et al. in CF patients [63]. The increase in bacteria capable of degrading xenobiotic compounds is probably due to the higher exposure to antibiotics and pharmacological treatments, recurrent in CF patients [65]. An enhanced ability of CF patients’ GM to metabolize proteins was highlighted by the increase in amino acid catabolism (e.g., valine, leucine, isoleucine, and lysine degradation) prediction. Indeed, the increase in protein catabolism in CF individuals has been well documented, probably due to the breakdown of both cellular and connective tissue proteins, which is related to the degree of impaired lung function and to the systemic inflammatory response [66]. Moreover, also valine, leucine, isoleucine, lysine, phenylalanine, tyrosine, and tryptophan biosynthesis pathways were linked to putative probiotics in HC. Our results nicely agree with the findings of a study carried out by Palmer et al., in which 11 genes involved in branched-chain and aromatic amino acid catabolism were highly upregulated in CF patients' sputum, while genes involved in the biosynthesis of these amino acids were repressed [67].

Moreover, the flagellar assembly pathway was associated with putative probiotics in CF. Bacterial flagellin is classified as a potent mediator of virulence of Gram-negative bacteria. Recurrent infections caused by Gram-negative strains could be linked to this inferred pathway [68]. 

Finally, the prediction of lipoic acid metabolism and folate biosynthesis pathways were associated with CF patterns. Consistently, Quinn and colleagues reported the abundance of lipoic acid metabolism in the lung of CF patients [65]. Lipoic acid is an antioxidant and a potent quencher of reactive oxygen species (ROS) [69], and it is used as a metabolic cofactor by Proteobacteria, Gram-positive bacteria, and *Pseudomonas aeruginosa* [69,70]. Quinn reported also high abundance of folate synthesis in CF patients [65]. Sulfonamides, such as sulfamethoxazole and trimethoprim, are antibiotics commonly used to treat CF infections that target microbial enzymes required for folate biosynthesis [71]. Prolonged exposure to sulfonamides may select microbes with multiple copies of these genes to overcome the drug’s effect on folate synthesis [72].

## 5. Conclusions

In conclusion, patients with CF usually have an abnormal intestinal microbiota and dysregulated immune mediators resulting from a massive exposure to antibiotics. Probiotics as immunomodulatory and anti-inflammatory substances are considered to improve both the clinical and the biochemical intestinal and pulmonary function in CF patients. The results reported in this study may point out new putative probiotic species on the basis of the GM differential profiles and predicted metabolic pathways of CF patients compared to HC. 

On the basis of our data, we speculate that some putative probiotic species, such as *B. longum*, *E. rectale*, *E. limosum*, *E. siraeum*, *L. sanfranciscensis*, *L. fermentum*, *B. pseudocatenulatum*, *B. bifidum,* and *F. prausnitzii* and their produced metabolites may have a protective role against CF disorders. Nevertheless, further in vitro studies and clinical trials should focus on these probiotics to assess whether the administration of selected strains, alone or in combination, may improve the quality of life and the clinical management of CF patients.

## Figures and Tables

**Table 1:** Cystic fibrosis (CF) Patients and healthy controls (HC) features.

Subjects	Males	Mean Age	Mean W/L or BMI Z-Score *	Pancreatic Insufficiency: Yes/Not	Mean Value of Sweat Test	Chronic Use of Antibiotic: Yes/No	Disease Severity: Mild/Severe
CF	11/28 (39%)	3.5	±0.9	22/6 79/21 (%)	93	12/16 43/57 (%)	4/24 14/86 (%)
HC	20/31 (64.5%)	3.06	±0.51	nda **	nda	nda	nda

* BMI/Z-Score: body mass index (BMI) (for patients over 2 years of age) or Z-score (Weight/Length (W/L) (for patients under 2 years of age); ** nda: no data associated.

**Table 2:** List of 20 bacteria prevalently associated with the GM profile of HC and CF patients. These OTUs were chosen for their putative probiotic role.

Bacteria	Group of Subjects
*Bifidobacterium bifidum*	HC
*Bifidobacterium longum*	
*Bifidobacterium pseudocatenulatum*	
*Faecalibacterium prausnitzii*	
*Lactobacillus fermentum*	
*Lactobacillus sanfranciscensis*	
*Eubacterium siraeum*	
*Eubacterium rectale*	
*Eubacterium limosum*	
*Bifidobacterium breve*	CF
*Bifidobacterium dentium*	
*Lactobacillus crispatus*	
*Lactobacillus mucosae*	
*Lactobacillus pentosus*	
*Lactobacillus pontis*	
*Lactobacillus reuteri*	
*Lactobacillus vaginalis*	
*Lactobacillus zeae*	
*Eubacterium biforme*	
*Eubacterium dolichum*	

**Table 3:** Kyoto Encyclopedia of Genes and Genomes (KEGG) pathways associated with HC and CF subjects.

KEGG Pathways	Class *	Subclass	Group	KEGG Pathways	Class	Subclass	Group
Carbon fixation in photosynthetic organisms	1	Energy metabolism	HC	Lysine degradation	1	Amino acid metabolism	CF
Alanine aspartate and glutamate metabolism		Amino acid metabolism		Phenylalanine metabolism			
Arginine and proline metabolism				Tryptophan metabolism			
Histidine metabolism				Tyrosine metabolism			
Lysine biosynthesis				Valine leucine and isoleucine degradation			
Phenylalanine tyrosine and tryptophan biosynthesis				Ascorbate and aldarate metabolism		Carbohydrate metabolism	
Valine leucine and isoleucine biosynthesis				Butanoate metabolism			
Flavonoid biosynthesis		Biosynthesis of other secondary metabolites		Citrate cycle TCA cycle			
Streptomycin biosynthesis				Carbon fixation pathways in prokaryotes		Energy metabolism	
C5 Branched dibasic acid metabolism		Carbohydrate metabolism		Sulfur metabolism			
Pentose phosphate pathway							
Propanoate metabolism				Fatty acid metabolism		Lipid metabolism	
Starch and sucrose metabolism							
Methane metabolism		Energy metabolism		Synthesis and degradation of ketone bodies			
Photosynthesis							
N Glycan biosynthesis		Glycan biosynthesis and metabolism		Folate biosynthesis		Metabolism of cofactors and vitamins	
Other glycan degradation				Lipoic acid metabolism			
Primary bile acid biosynthesis		Lipid metabolism					
Secondary bile acid biosynthesis				Ubiquinone and other terpenoid quinone biosynthesis			
Sphingolipid metabolism							
Biotin metabolism		Metabolism of cofactors and vitamins		Glutathione metabolism		Metabolism of other amino acids	
Pantothenate and CoA biosynthesis							
Riboflavin metabolism				Taurine and hypotaurine metabolism			
Vitamin B6 metabolism							
Cyanoamino acid metabolism		Metabolism of other amino acids		Biosynthesis of siderophore group nonribosomal peptides		Metabolism of terpenoids and polyketides	
D Alanine metabolism							
Biosynthesis of vancomycin group antibiotics		Metabolism of terpenoids and polyketides		Aminobenzoate degradation		Xenobiotics biodegradation and metabolism	
Polyketide sugar unit biosynthesis				Benzoate degradation			
Atrazine degradation		Xenobiotics biodegradation and metabolism		Dioxin degradation			
Protein processing in endoplasmic reticulum	2	Folding, sorting and degradation		Ethylbenzene degradation			
RNA degradation				Fluorobenzoate degradation			
Base excision repair		Replication and repair		Xylene degradation			
Non homologous end joining				Sulfur relay system	2	Folding, sorting and degradation	
Basal transcription factors		Transcription					
Aminoacyl tRNA biosynthesis		Translation		Two component system	3	Signal transduction	
Insulin signaling pathway	5	Endocrine system		Flagellar assembly	4	Cell motility	
Nucleotide oligomerization domain (NOD) like receptor signaling pathway		Immune system		Peroxisome		Transport and catabolism	
Amoebiasis	6	Infectious diseases		Chagas disease American trypanosomiasis	6	Infectious diseases	
Epithelial cell signaling in *Helicobacter pylori* infection							

* Class: 1. Metabolism; 2. Genetic Information Processing; 3. Environmental Information Processing; 4. Cellular Processes; 5. Organismal Systems; 6. Human Diseases.
